# Genetic Heterogeneity of* Alicyclobacillus* Strains Revealed by RFLP Analysis of* vdc* Region and* rpoB* Gene

**DOI:** 10.1155/2018/9608756

**Published:** 2018-11-01

**Authors:** Agnieszka Dekowska, Jolanta Niezgoda, Barbara Sokołowska

**Affiliations:** Prof. Waclaw Dabrowski Institute of Agricultural and Food Biotechnology, 36 Rakowiecka Street, 02-532 Warsaw, Poland

## Abstract

PCR-RFLP targeting of the 16S rDNA and* rpoB* genes, as well as the* vdc* region, was applied to identify and differentiate between the spoilage and non-spoilage* Alicyclobacillus* species. Eight reference strains and 75 strains isolated from spoiled juices, juice concentrates, drinks, its intermediates, and fresh apples were subject to study. Hin6I restriction patterns of the 16S rDNA gene enabled distinguishing between all the species analyzed, while the* rpoB* gene and* vdc* gene cluster analysis also revealed that there were two major types among the* A. acidoterrestris* isolates, one similar to the reference strain* A. acidoterrestris* DSM 2498, and the other similar to the reference strain* A. acidoterrestris* ATCC 49025. Heterogeneity was also observed among the* A. acidocaldarius* isolates. RFLP analysis of the 16S rDNA and* rpoB* genes, as well as* vdc* region, can be used successfully in the identification and research of intraspecies heterogeneity of the* Alicyclobacillus* species.

## 1. Introduction

The contamination of fruit juices by* Alicyclobacillus* has recently become one of the most important issues in the juice and beverage industry. These acidophilic, thermophilic, and spore-forming bacteria are very hard to eliminate from contaminated drinks.

*Alicyclobacillus* are Gram positive, aerobic, soil borne bacteria that are able to grow within a range from pH 2.0 to 6.0 and at temperatures from 20 to 70°C [1–8]. The two main factors which prevent fruit products from spoilage with most other bacteria, which are thermal treatment and low pH values, are insufficient to eliminate* Alicyclobacillus*. The spores survive under typical pasteurization conditions and are able to germinate and grow in an acidic environment [9, 10]. Thermal treatment may even impel germination of the spores [11–13].

Despite being nonpathogenic [14], some* Alicyclobacillus* species may cause the spoilage of juices and juice-containing products such as nectars and beverages.* Alicyclobacillus* species have been found all across the world, and their presence has been detected on fruit surfaces [15], in juices produced from several fruits: citrus, apple, banana, berry, and stone fruits [1, 8, 10, 16–24], in canned tomatoes [25], and in drinks, for example ice tea, and isotonic drinks [17, 26]. The spoilage mainly manifests itself as the formation of a medical, antiseptic off-odour, from compounds produced by the bacteria. The main compound associated with spoilage is guaiacol, produced from vanillin and vanillic acid [27–29], but halophenols, 2,6-dibromophenol and 2,6-dichlorophenol, have also been reported as spoilage agents. Among the 22 currently known* Alicyclobacillus* species, five have been proven to produce an off-odour:* A. acidoterrestris, A. acidiphilus, A. pomorum, A. cycloheptanicus, *and* A. herbarius*. [1, 9, 14, 30–37].

The classic method for isolating and characterizing* Alicyclobacillus*, devised by IFU (Internationale Fruchtsaft Union), which is commonly used in the juice and beverage industry, takes about 15 days. If present in the tested sample, guaiacol can be detected using the peroxidase method [27, 38], by sensory tests ([9, 10, 39]; Siegmund and Pöllinger-Zierler, 2006) or instrumental methods, for example, HPLC or gas chromatography [28, 35, 40, 41].

The identification of the* Alicyclobacillus* species based on their ability to assimilate erythritol with acid production [19, 42] mainly allows differentiating between two species:* A. acidoterrestris* and* A. acidocaldarius*.

Since the classic microbiological* Alicyclobacillus* detecting methods are time consuming, alternative approaches have been adopted, like flow cytometry ([30], Pieper et al. 2006), Fourier transform infrared spectroscopy [43–45], and genetic methods. Except for RAPD-PCR, (Yamazaki et al., 1997; [46–49]), most of the genetic methods used in the studies on* Alicyclobacillus* target the rDNA operon. These include 16S rDNA and ITS region sequencing, 16S rDNA RFLP, Real-Time PCR, and LAMP-PCR of the 16S rDNA fragment ([1, 50]; Durak et al., 2002; [6, 7, 51–54]).

Guaiacol, the main spoilage agent, is produced by nonoxidative decarboxylation of vanillic acid. The ability to produce guaiacol is associated with presence of* vdc* gene cluster, consisting of three genes,* vdcB*,* vdcC,* and* vdcD*. Chow et al. [55] described* vdc* gene cluster in* Streptomyces* sp. Detection of the* vdc* genes of* A. acidoterrestris* using RT-PCR was described by Niwa and Kawamoto [38]. The* vdc* region sequence was published by Matsubara [56]. To date, there are no applications of* vdc* region analysis for any microorganism.* RpoB* gene, encoding the *β* subunit of bacterial RNA polymerase, is one of the single-copy housekeeping genes and is widely used in studies on bacterial taxonomy. These studies include PCR-RFLP analyses of* rpoB* gene fragments; however, so far, there are no reports on using* rpoB* gene analysis in research on of* Alicyclobacillus*.

Our study focuses on the use of* rpoB* gene,* vdc* region, and 16SrDNA gene as molecular markers for the identification and differentiation of* Alicyclobacillus*.

## 2. Materials and Methods

### 2.1. Sample Acquisition and Bacterial Strains

Seventy-five strains analyzed in this study were isolated from concentrated apple juice (47), concentrated cherry juice (4), fresh apples (3), concentrated strawberry juice (2), concentrated black currant juice (2), tomato juice (2), orange juice (3), cloudy (1) and clear (1) apple juice, apple beverage (1), concentrated beetroot juice (1), concentrated raspberry juice (1), concentrated orange juice (1), orange beverage (1), banana nectar (1), cherry puree (1), and intermediates used in beverage production (3). All the strains were isolated according to the method described in IFU no. 12 September 2004/March 2007.

Reference strains were obtained from the Leibniz Institute* DSMZ* – German Collection of Microorganisms and Cell Cultures (*Alicyclobacillus acidiphilus *DSM 14558;* Alicyclobacillus acidocaldarius* DSM 446;* Alicyclobacillus acidoterrestris* DSM 2498;* Alicyclobacillus herbarius *DSM 13609;* Alicyclobacillus hesperidum* DSM 12489), and from the American Type Culture Collection (ATCC) (*Alicyclobacillus acidoterrestris *ATCC 49025;* Geobacillus stearothermophilus* ATCC 7953;* Bacillus subtilis *ATCC 6655). Also,* Alicyclobacillus acidocaldarius* A1 from our own library collection was used as a reference strain, after biochemical and 16S rDNA sequencing confirmation.

### 2.2. Biochemical Tests

The strains were checked for erythritol utilization and guaiacol production. The ability of erythritol utilization was tested by plating the cultures on agar containing 1% erythritol and bromophenol blue as an indicator [42]. The ability to produce guaiacol was tested using the peroxidase method [27].

### 2.3. DNA Isolation

Selected* Alicyclobacillus* strains were cultured in BAT medium (pH 4.0±0.2) at 45°C for 2–3 days.* Bacillus subtilis* was cultured in TSB medium (pH 7.1±0.2) at 30°C for 2 days, and* Geobacillus stearothermophilus* was cultured in TSB medium at 45°C for 2 days.

Bacterial chromosomal DNA was purified using a Genomic Mini Kit (A&A Biotechnology), following the manufacturer's instructions.

### 2.4. Primer Designing and Amplification

All PCR reactions were performed using a Pequstar 2x Gradient thermocycler (Pequlab).

#### 2.4.1. 16S rDNA Amplification

A fragment of the 16S rDNA gene was amplified using universal primers, similar to that used by Wang et al., 2010 [57]. The primer sequences were 8F (5′– AGAGTTTGATCCTGGCTCAG),* E. coli* positions 8-27 and 1512R, shortened by 1 nucleotide at 3′ end (5′ – ACGGCTACCTTGTTACGACT),* E. coli* positions 1512–1493. The size of the amplification product was 1495 bp.

PCR reactions were performed in a total volume of 50 *μ*l, containing 5 ng of the template DNA, 50 pM of each of the primers, and 25 *μ*l of the DreamTaq™ Green PCR Master Mix (Thermo Scientific). PCR was performed under the following conditions: initial denaturation at 94°C for 2 min, 40 cycles of denaturation at 94°C for 30 sec, annealing at 51°C for 35 sec, elongation 72°C for 1 min 40 sec, and the final elongation at 72°C for 2 min.

#### 2.4.2. *vdc* Region Amplification

**Figure 1:**
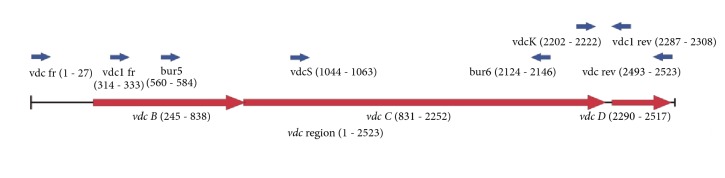
*Vdc* region, positions of genetic elements and primers.

The* vdc* gene cluster was amplified using primers vdc fr (5′ – CTGTTGGCTCAATGGCGGCTGAGCGAT), vdc rev (5′ – TTATCAGCGGTTTATCCGCGGTGGAACAGTC), vdc1 fr (5′ – AACGACGCAGGTGTGGAAAC), vdc1 rev (5′ – AGCGTGGGCAAGTTGTCATGTG), vdc K (5′ – TTGGCAACGGAGAAGTGGGAG) and vdc S (5′ – AATCACGCGCTGATGATGGG). The 1586 fragment of* vdc* region, containing fragments of* vdcB* and* vdcC* genes, was used as a template for PCR-RFLP and was amplified using the primers Bur 5 (5′ GCCGACGTGATGCTCAARGAGCGCA) and Bur 6 (5′ GTSGCRTCGAGAATCATCTTGTG). The primers were designed based on a comparison of the raw genome sequences derived from* Alicyclobacillus acidoterrestris* ATCC 49025 ([58]; GenBank number AURB01000113.1), and* Alicyclobacillus herbarius* DSM 13609 (GenBank number AUMH01000032.1), the sequence published by Matsubara (GenBank number BD187778.1), and sequences obtained from several* Alicyclobacillus acidoterrestris* strains analyzed in this study. Sequence alignments were performed using the Serial Cloner program. The positions and directions of the* vdc* primers are described in Table 1 and shown in Figure 1.

The 2523 bp vdc fr – vdc rev amplification product contained the whole* vdc* region. The size of the Bur5-Bur6 amplification product was 1586 bp. PCR reactions were performed in a total volume of 50 *μ*l, containing 2.5 ng of the template DNA, 5 pM of each of the primers, and 25 *μ*l of the DreamTaq™ Green PCR Master Mix (Thermo Scientific). PCR was performed under the following conditions: initial denaturation at 94°C for 2 min, 30 cycles of denaturation at 94°C for 30 sec, annealing at 59°C for 50 sec, elongation 72°C for 1 min 40 sec, and the final elongation at 72°C for 5 min.

#### 2.4.3. *rpoB* Gene Amplification

A fragment of* rpoB* gene was amplified using Gru3–Gru6 primers. The primers were designed based on a comparison of the* rpoB* gene sequences of* Alicyclobacillus acidocaldarius*,* Bacillus subtilis*, and* Geobacillus stearothermophilus*, as well as sequences derived from* A. acidoterrestris* 49025,* A. hesperidum* URH17-3-68, and* A. herbarius* DSM 13609 raw genomic sequences. Gru5 and Gru6 primers are nondegenerate versions of the Gru3 and Gru4 primers, respectively. The primer sequences were Gru3 (CGYGACGTDCACTAYTCBCACTA), Gru4 (5′ – GCCCANACYTCCATCTCRCCRAA) Gru5 (5′ – CGCGACGTACACTATTCGCACTA), and Gru6 (5′ – GCCCAAACCTCCATCTCACCAAA). The size of the amplification product was 1735 bp. PCR reactions were performed in a total volume of 50 *μ*l, containing 30 ng of the template DNA, 40 pM of each of the primers, and 25 *μ*l of the DreamTaq™ Green PCR Master Mix (Thermo Scientific). PCR was performed under the following conditions: initial denaturation at 94°C for 2 min, 35 cycles of denaturation at 94°C for 30 sec, annealing at 57°C (for the Bur5 and Bur6 primer pairs) or at 59°C (for the Bur3 and Bur4 primers) for 35 sec, elongation 72°C for 1 min 45 sec, and the final elongation at 72°C for 5 min.

### 2.5. PCR-RFLP

5-15 *μ*l of the PCR products were digested with BsuRI, Hin6I, and HphI (Thermo Scientific) in 20 *μ*l volumes. The samples were incubated at 37°C for 1-2 hours, and the enzymes were then inactivated with ten-minute incubation at 80°C. The digests were analyzed on 2.5–3% agarose gel.

### 2.6. DNA Sequencing

DNA samples were sequenced using 8F and shortened 1512R primers for the 16S rDNA gene; vdc fr, vdc rev, vdc1 fr, vdc1 rev, Bur5, Bur6, and two additional primers to fill the gaps, vdcS (5′ – AATCACGCGCTGATGATGGG) and vdcK (5′ – TTGGCAACGGAGAAGTGGGAG) for the* vdc* region; and Gru3 – Gru6 for the* rpoB *gene.

The contig assemblies, sequence alignments, and phylogenetic analysis were performed using Serial Cloner software (SerialBasic) and Clustal Omega. The sequences were compared to the GenBank sequences database using BLAST tools.

## 3. Results

### 3.1. RFLP Analysis of 16S rDNA Fragment

The 1495 bp fragment of the 16S rDNA gene, amplified using 8F and shortened 1512R primers, was digested by BsuRI, Hin6I, and HphI.

**Figure 2:**
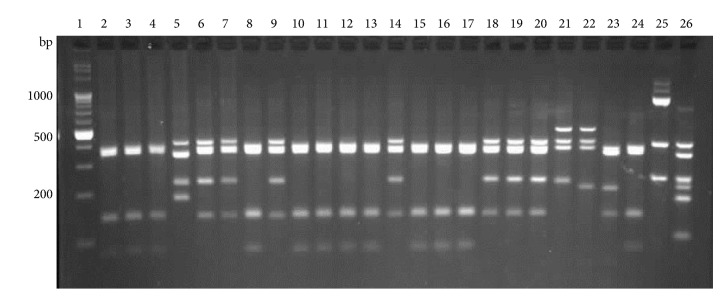
Restriction patterns of a 1495 bp fragment of the 16S rDNA gene, digested with Hin6I. Lanes 6, 7, 9, 14, and 18,* A. acidoterrestris*; 2, 3, 4, 8, 10, 11, 12, 13, 15, 16, and 17,* A. acidocaldarius*; 5,* Brevibacillus agri*; 19* A. acidoterrestris* DSM 2498; 20,* A. acidoterrestris* ATCC49025; 21,* A. acidiphilus*; 22,* A*.* hesperidum*; 23,* A*.* herbarius*; 24,* A. acidocaldarius*; 25,* B. subtilis*; 26,* G. stearothermophilus*; lane 1, molecular marker.

While BsuRI and HphI digestions did not allow to distinguish between all the species analyzed, the patterns obtained by Hin6I digestion were species specific (Figure 2).

All analyzed isolates with their features and RFLP profiles are described in Table 2.

### 3.2. RFLP Analysis of the* vdc* Region Fragment

**Figure 3:**
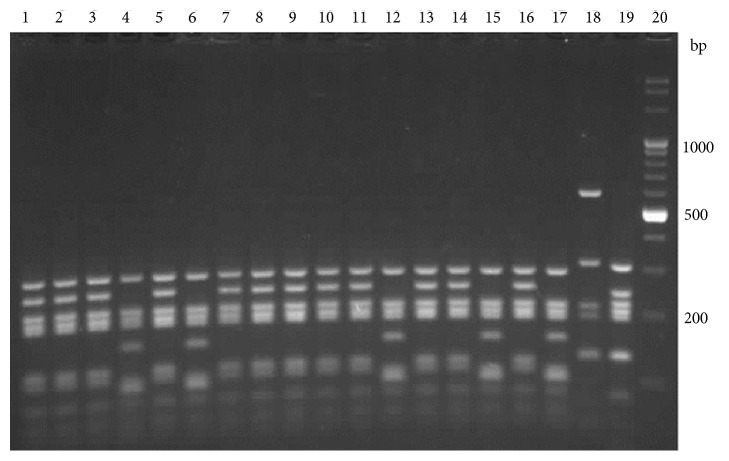
Restriction patterns of a 1586 bp fragment of the* vdc* region, digested with BsuRI. Lanes 1–3, 5, 7–11, 13, and 14,* A. acidoterrestris* type I; 4, 6, 12, and 15,* A. acidoterrestris* type II; 16* A. acidoterrestris* DSM 2498; 17,* A. acidoterrestris* ATCC49025; 18,* A. acidiphilus*; 19,* A*.* herbarius*; 20, molecular marker.

**Figure 4:**
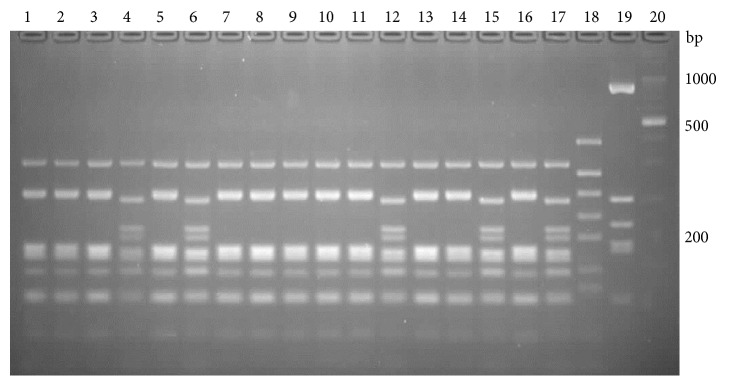
Restriction patterns of a 1586 bp fragment of the* vdc* region, digested with Hin6I. Lanes 1–3, 5, 7–11, 13, and 14,* A. acidoterrestris* type I; 4, 6, 12, and 15,* A. acidoterrestris* type II; 16* A. acidoterrestris* DSM 2498; 17,* A. acidoterrestris* ATCC49025; 18,* A. acidiphilus *DSM 14558; 19,* A*.* herbarius *DSM 13609; lane 20, molecular marker.

**Figure 5:**
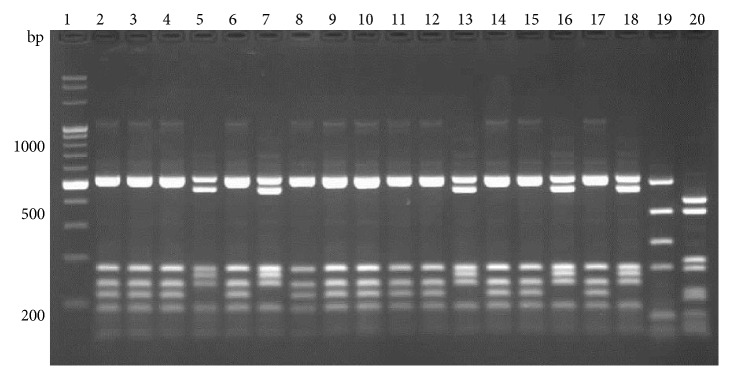
Restriction patterns of a 1586 bp fragment of the* vdc* region, digested with HphI. Lanes 2–4, 6, 8–12, 14, and 15,* A. acidoterrestris* type I; 5, 7, 13, and 16,* A. acidoterrestris* type II; 17* A. acidoterrestris* DSM 2498; 18,* A. acidoterrestris* ATCC49025; 19,* A. acidiphilus*; 20,* A*.* herbarius*; lane 1, molecular marker.

The fragment of* vdc* region was amplified using Bur5 and Bur6 primers. The product was a single band of 1586 bp and was observed only for guaiacol producing strains:* A. acidoterrestris*,* A. acidophilus*, and* A. herbarius*. The PCR product was digested by BsuRI (Figure 3), Hin6I (Figure 4), and HphI (Figure 5).

BsuRI and Hin6I RFLP patterns enabled distinguishing between all the species analyzed, and divided the* A. acidoterrestris* group into two clusters. Type I pattern was identical to the pattern given by* A. acidoterrestris* DSM 2498, and type II pattern was identical to* A. acidoterrestris *ATCC 49025. The HphI patterns confirm this rule with the exception of one strain, which represented the type I pattern in the BsuRI and Hin6I analysis.

### 3.3. RFLP Analysis of the* rpoB* Gene Fragment

The 1735 bp fragment of the* rpoB* gene was amplified using Gru3 and Gru4, or Gru5 and Gru6 primers. Gru3 and Gru4 primers were degenerated versions of Gru5 and Gru6 (respectively) and were used for amplification of isolates other than* A. acidoterrestris*.* A acidoterrestris* isolates were amplified either with Gru3–Gru4, or Gru5–Gru6 primers, and the Gru5–Gru6 primer pair was chosen due to the better efficiency of the reaction. Considering the individual isolates, the RFLP patterns were identical for both pairs of primers.

**Figure 6:**
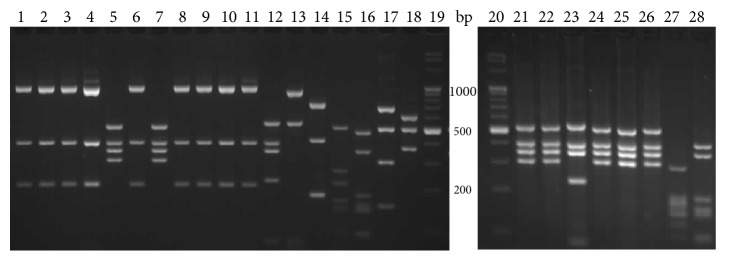
Restriction patterns of a 1735 bp fragment of the* rpoB* gene, digested with BsuRI. Lanes 1–4, 6, and 8–10,* A. acidoterrestris* type I; 5, 7, 21, 22, and 24-26,* A. acidoterrestris* type II; 23,* A. acidoterrestris* type IIA; 27,* A. acidocaldarius* type II, 28,* A. acidocaldarius *type IA; 11,* A. acidoterrestris* DSM 2498; 12,* A. acidoterrestris* ATCC49025; 13,* A. acidiphilus*; 14,* A*.* hesperidum*; 15,* A*.* herbarius*; 16,* A. acidocaldarius* A1; 17,* B. subtilis*; 18,* G. stearothermophilus*; lane 19, molecular marker.

**Figure 7:**
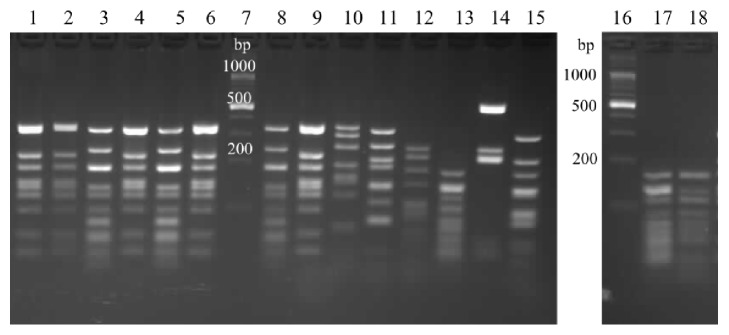
Restriction patterns of a 1735 bp fragment of* rpoB* gene, digested with Hin6I. Lanes 3 and 5,* A. acidoterrestris* type I; 1, 2, 4, and 6,* A. acidoterrestris* type II; 17,* A. acidocaldarius* type I; 18,* A. acidocaldarius* type II; 8,* A. acidoterrestris* DSM 2498; 9,* A. acidoterrestris* ATCC49025; 10,* A. acidiphilus*; 11,* A*.* hesperidum*; 12,* A*.* herbarius*; 13,* A. acidocaldarius* A1; 14,* B. subtilis*; 15,* G. stearothermophilus*; lanes 7 and 16, molecular marker.

**Figure 8:**
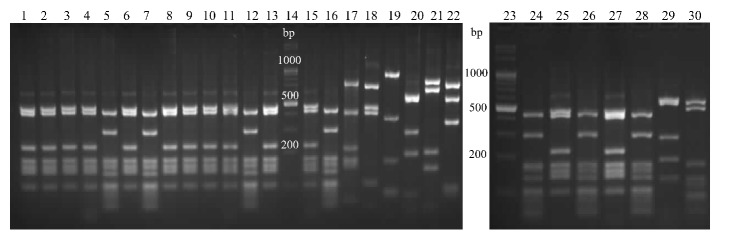
Restriction patterns of a 1735 bp fragment of* rpoB* gene, digested with HphI. Lanes 1-4, 6, 8-11, 13, 25, and 27,* A. acidoterrestris* I; 5, 7, 12, 26, and 28,* A. acidoterrestris* II; 24,* A. acidoterrestris* IIA; 29,* A. acidocaldarius* IA; 30, A. acidocaldarius type II; 15,* A. acidoterrestris* DSM 2498; 16,* A. acidoterrestris* ATCC49025; 17,* A. acidiphilus*; 18,* A*.* hesperidum*; 19,* A*.* herbarius*; 20,* A. acidocaldarius *A1; 21,* B. subtilis*; 22,* G. stearothermophilus*; lanes 14 and 23, molecular marker.

The patterns obtained by all of the nucleases used (Figures 6–8) enabled distinguishing between the species analyzed. With exception of two strains, the BsuRI patterns divided the* A. acidoterrestris* group into two clusters, analogical to the clusters revealed by the* vdc* region analysis. The pattern (IIA) is shown by two exceptional strains, most resembling the type II pattern of* A. acidoterrestris*, and those strains were classified as type II in other analyses. The reference strain ATCC 49025 shows this type of pattern. The* A. acidocaldarius* group was represented by three types of patterns, two of them resembling each other.* A acidocaldarius *DSM 446 belongs to the cluster II of* A. acidocaldarius* group, while* A. acidocaldarius* A1 was assigned to cluster I.

For Hin6I there were two types of patterns for the* A. acidoterrestris* and* A. acidocaldarius*.* A. acidoterrestris *DSM 2498 belongs to the cluster I, while* A. acidoterrestris* ATCC 49025 belongs to the cluster II*. A. acidocaldarius* DSM 446 represents cluster II of the* A. acidocaldarius group*, while* A. acidocaldarius* A1 represents cluster I.

HphI endonuclease produces two types of patterns for* A. acidoterrestris*, analogically as before, and three types of patterns for* A. acidocaldarius*. One sample of* A. acidoterrestris *cluster II has a slightly different pattern and has been classified as type II in other analyses. Two of the three patterns of* A. acidocaldarius* differ with only one band.* A. acidocaldarius* DSM 446 represents cluster II of the* A. acidocaldarius group*, while* A. acidocaldarius* A1 represents cluster I.

### 3.4. DNA Sequencing

DNA sequencing was performed for selected strains. Sequencing of the 16S rDNA gene was performed to confirm the proper identification of the isolates and to identify the non-*Alicyclobacillus* isolates. The 16S rDNA sequence of strains 31 and 34, classified as type I, showed the greatest similarity to* A. acidoterrestris* DSM 2498, also classified as type I. The sequences of strains 33, 51, 52, 53, 55, and 56, classified as type II, showed the greatest similarity to* A. acidoterrestris* ATCC 49025, also classified as type II. The sequence of strain 41, classified as type II, showed the greatest similarity to* A. acidoterrestris *DSM 3922.

**Figure 9:**
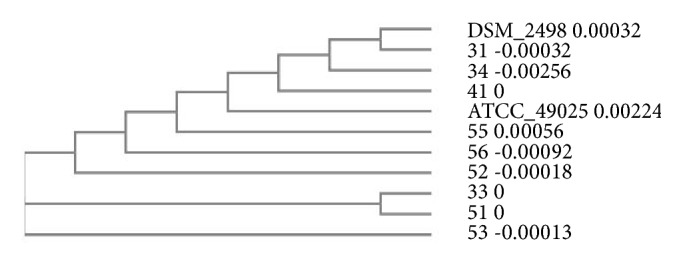
Phylogenetic tree of partial 16S rRNA sequence of 11* A. acidoterrestris* isolates. The tree was constructed by neighbor joining method using Clustal Omega software.

Figure 9 shows the phylogenetic tree constructed from the 16S rDNA sequences of analyzed strains of* A. acidoterrestris*. The sequence analysis indicates that* A. acidoterrestris* DSM 2498 and two other type I strains are closely related and it shows greater variation within class II strains. The isolates selected for sequencing represent both groups and different sources which they were isolated from: concentrated apple juice (isolates 33 and 41), fresh apples (isolate 34), concentrated blackcurrant juice (isolate 31), concentrated raspberry juice (isolate 56), concentrated beetroot juice (isolate 51), and concentrated cherry juice (isolates 52, 53, and 55). The sequence analysis shows no apparent correlation between diversity of the 16S rDNA sequence and the sources of the isolates.

Five whole* vdc* gene clusters were sequenced. Two of them were isolated from the reference strains,* A. acidoterrestris* DSM 2498, and* A. acidoterrestris* ATCC 49025. 2416 bp fragments of the* vdc* regions were aligned. The* vdc* region sequence of strains 15 and 34, classified as type I, showed 99.9% identity with the* vdc* sequence of* A. acidoterrestris *DSM 2498; the sequence of strain 41, classified as type II, showed 99.3% identity with the* vdc* sequence* A. acidoterrestris* ATCC 49025.* Vdc* sequences of* A. acidoterrestris *DSM 2498 and* A. acidoterrestris *DSM 49025 showed 94.5% identity. For the last pair, protein sequences of the gene products showed 98.5% positives and 96.5% identities for VdcB, 99.4% and 98.9% for VdcC, and 100% and 97.4% for VdcD.

*RpoB* gene sequencing was performed to confirm that both primer pairs, Gru3–Gru4 and Gru5–Gru6, enabled to amplify the correct DNA fragment and that degeneration of the primers did not affect the sequence specificity, although the degenerated primers produced significantly lower amount of PCR product. All of the sequences obtained showed greatest similarity to the appropriate* rpoB* genes.

The GenBank accession numbers are KX371237-KX371249 for 16S rDNA sequences; KX453673-KX453677 for full* vdc* region sequences; KX453678 and KX453679 for partial* vdc* sequences of* A. herbarius* and* A. acidiphilus*, respectively; and KX453680- KX453682 for partial* rpoB* sequences.

## 4. Discussion

Among more than one thousand samples of concentrated apple juice tested in our laboratory between 2004 and 2012, 67% was contaminated with* Alicyclobacillus* sp., and 31% of the isolates were identified as* A. acidoterrestris *[59]. The statistics show that* Alicyclobacillus* spoilage is still a major concern in the fruit processing industry.

In this study, 75 guaiacol producing and non-guaiacol producing strains were isolated from various fruit juices, concentrated fruit juices, and fresh apples. Additionally, 8 reference strains were used. The isolates and reference strains were examined using classic methods, and PCR-RFLP focusing on 16S rDNA and* rpoB* gene fragments as well as the* vdc *region fragment. For selected strains, DNA sequencing of the 16S rDNA gene was performed. Sixty of the isolates analyzed were identified as* A. acidoterrestris*, 12 as* A. acidocaldarius*, one as* Brevibacillus agri*, and two as* Bacillus ginsengihumi.* Four of the isolates, which gave ambiguous results during classic identification (nontypical colour on an erythritol medium or lack of growth at 65°C connected with lack of guaiacol production), were identified by genetic methods as* A. acidoterrestris* or* A. acidocaldarius*.* A. acidoterrestris* isolates were grouped in two major clusters. 27 of the isolates belonged to the cluster I, and 33 to the cluster II. Also,* A. acidocaldarius* isolates were grouped into two clusters, but showed more intracluster diversity.

These results support the observations made by Osopale at al. [60] and Durak et al. [23] who also reported two genetic clusters among analyzed* A. acidoterrestris* samples, by RAPD analysis,and by 16S rDNA sequencing, respectively.

Most of the strains analyzed in this study were isolated from concentrated apple juice, as this is the main subject of the* Alicyclobacillus* focused screening made or outsourced by polish fruit industry; however, it is one of the main subjects of similar screenings performed by fruit industry worldwide.* A. acidoterrestris* representing both clusters have been found in concentrated apple juice. For other sources, three* A. acidoterrestris* cluster II isolates have been found in orange juices, two cluster I in concentrated black currant juice, and three cluster II in concentrated cherry juice; however, these are only single observations and further research is needed to establish if* A. acidoterrestris* strains representing both clusters are found in other juices.

Genetic methods are broadly used in the detection, characterization, and differentiation of microorganisms. The application of PCR-RFLP in the characterization of* Alicyclobacillus* and other thermoacidophilic bacteria isolated from the apple juice processing environment has been described by Chen et al. [52], although only the 16S rDNA gene was the subject of this study and the Hin6I enzyme was not used.* RpoB* gene has never been subjected to PCR-RFLP or any other genetic analysis of* Alicyclobacillus* so far, although the value of this gene in taxonomy has been confirmed by many studies on other microorganisms. To date, there are no reports on the use of the* vdc* gene cluster in taxonomic studies of microorganisms, although its sequence may be proven valuable for research both on the vectors and diversity and evolution of the element itself. Hin6I restriction patterns for 16S rDNA were sufficient to differentiate between all the species analyzed, but provided no closer data on intraspecies diversity. The diversity was revealed both by the* rpoB* gene and* vdc* region RFLP analyses.

*Vdc* gene cluster of* Streptomyces* sp., described by Chow et al. [55], consists of three genes:* vdcB* (0.6kb),* vdcC* (1.4 kb), and* vdcD* (0.2 kb), transcribed as single, policistronic mRNA molecule. All three genes were essential to produce guaiacol from vanillic acid. Niwa and Kawamoto [38] described analogical gene cluster in* A. acidoterrestris*, consisting of ORF1 (597 bp), ORF2 (1425 bp), and ORF3 (321 bp). Three respective open reading frames were found in all five analyzed* vdc* sequences.

*Alicyclobacillus acidoterrestris* RFLP patterns of both the* rpoB* gene and* vdc* region showed consistently that there were two major types among the isolates, one similar to the reference strain* A. acidoterrestris* DSM 2498, and the other similar to the reference strain* A. acidoterrestris* ATCC 49025. Obtaining such consistent data concerning two genetic elements of distant function reveals that there is deeper intraspecies genetic diversity in the* A. acidoterrestris* species. This division may be considered when examining other features of* Alicyclobacillus* such as susceptibility to temperature or high hydrostatic pressure, in order to establish if there are any differences between the groups.

Both the* rpoB* gene and* vdc* region seem to be single-copy genetic elements and showed no signs of intragenomic heterogeneity, which often makes the RFLP comparison of rDNA genes harder to perform.

The sequence analysis of the 16S rDNA fragment of selected isolates shows no apparent correlation between diversity of the 16S rDNA sequence and the sources which the strains were isolated from.

In conclusion, the application of PCR-RFLP has been proven to be a fast and reliable method for* Alicyclobacillus* identification and differentiation. The method is also technically less difficult than most of other molecular techniques. Two major groups of* A. acidoterrestris *have been identified. The primers designed for this study could be useful in further research on* Alicyclobacillus*.

## Figures and Tables

**Table 1:** The positions and directions of vdc primers.

Primer name	Primer position (bp)	Primer direction
vdc fr	1-27	forward

vdc rev	2493-2523	reverse

vdc1 fr	314-333	forward

vdc1 rev	2287-2308	reverse

vdc K	2202-2222	forward

vdc S	1044-1063	forward

Bur5	560-584	forward

Bur6	2124-2146	reverse

**Table 2:** List of isolates and their RFLP profiles. AAT, *A. acidoterrestris*; AAC, *A. acidocaldarius*; BA, *Brevibacillus agri*; BG, *bacillus ginsengihumi*.

Isolate	Source	Guaiacol production	Erythritol utilization	RFLP analysis						
				16S/ Hin61	*vdc*/ BsuRI	*vdc*/ Hin61	*vdc*/ HphI	*rpoB*/ BsuRI	*rpoB*/ Hin61	*rpoB*/ HphI
1	concentrated apple juice	yes	yes	AAT	AAT(I)	AAT(I)	AAT(I)	AAT(I)	AAT(I)	AAT(I)
2	concentrated apple juice	yes	yes	AAT	AAT(I)	AAT(I)	AAT(I)	AAT(I)	AAT(I)	AAT(I)
3	intermediate for beverage production	yes	yes	AAT	AAT(I)	AAT(I)	AAT(I)	AAT(I)	AAT(I)	AAT(I)
4	intermediate for beverage production	no	no	AAC	-	-	-	AAC(I)	AAC(I)	AAC(I)
5	concentrated apple juice	yes	yes	AAT	AAT(I)	AAT(I)	AAT(I)	AAT(I)	AAT(I)	AAT(I)
6	concentrated apple juice	yes	yes	AAT	AAT(II)	AAT(II)	AAT(II)	AAT(II)	AAT(II)	AAT(II)
7	concentrated apple juice	yes	yes	AAT	AAT(I)	AAT(I)	AAT(I)	AAT(I)	AAT(I)	AAT(I)
8	concentrated apple juice	yes	yes	AAT	AAT(II)	AAT(II)	AAT(II)	AAT(II)	AAT(II)	AAT(II)
9	orange beverage	yes	yes	AAT	AAT(I)	AAT(I)	AAT(I)	AAT(I)	AAT(I)	AAT(I)
10	concentrated apple juice	yes	yes	AAT	AAT(I)	AAT(I)	AAT(I)	AAT(I)	AAT(I)	AAT(I)
11	concentrated apple juice	yes	yes	AAT	AAT(I)	AAT(I)	AAT(I)	AAT(I)	AAT(I)	AAT(I)
12	concentrated apple juice	yes	yes	AAT	AAT(I)	AAT(I)	AAT(I)	AAT(I)	AAT(I)	AAT(I)
13	concentrated apple juice	yes	yes	AAT	AAT(I)	AAT(I)	AAT(I)	AAT(I)	AAT(I)	AAT(I)
14	concentrated apple juice	yes	yes	AAT	AAT(II)	AAT(II)	AAT(II)	AAT(II)	AAT(II)	AAT(II)
15	concentrated apple juice	yes	yes	AAT	AAT(I)	AAT(I)	AAT(I)	AAT(I)	AAT(I)	AAT(I)
16	concentrated apple juice	yes	non typical	AAT	AAT(I)	AAT(I)	AAT(I)	AAT(I)	AAT(I)	AAT(I)
17	banana nectar	yes	yes	AAT	AAT(I)	AAT(I)	AAT(I)	AAT(I)	AAT(I)	AAT(I)
18	orange juice	yes	yes	AAT	AAT(II)	AAT(II)	AAT(II)	AAT(II)	AAT(II)	AAT(II)
19	concentrated orange juice	yes	yes	AAT	AAT(II)	AAT(II)	AAT(II)	AAT(II)	AAT(II)	AAT(II)
20	concentrated apple juice	yes	yes	AAT	AAT(II)	AAT(II)	AAT(II)	AAT(II)	AAT(II)	AAT(II)
21	orange juice	yes	yes	AAT	AAT(II)	AAT(II)	AAT(II)	AAT(II)	AAT(II)	AAT(II)
22	concentrated apple juice	yes	yes	AAT	AAT(II)	AAT(II)	AAT(II)	AAT(II)	AAT(II)	AAT(II)
23	concentrated apple juice	yes	yes	AAT	AAT(I)	AAT(I)	AAT(I)	AAT(I)	AAT(I)	AAT(I)
24	concentrated apple juice	yes	yes	AAT	AAT(I)	AAT(I)	AAT(I)	AAT(I)	AAT(I)	AAT(I)
25	fresh apples	yes	yes	AAT	AAT(II)	AAT(II)	AAT(II)	AAT(II)	AAT(II)	AAT(II)
26	concentrated apple juice	yes	yes	AAT	AAT(II)	AAT(II)	AAT(II)	AAT(II)	AAT(II)	AAT(II)
27	concentrated apple juice	yes	yes	AAT	AAT(II)	AAT(II)	AAT(II)	AAT(II)	AAT(II)	AAT(II)
28	concentrated apple juice	yes	yes	AAT	AAT(II)	AAT(II)	AAT(II)	AAT(II)	AAT(II)	AAT(II)
29	concentrated apple juice	yes	yes	AAT	AAT(II)	AAT(II)	AAT(II)	AAT(II)	AAT(II)	AAT(II)
30	concentrated apple juice	yes	yes	AAT	AAT(II)	AAT(II)	AAT(II)	AAT(II)	AAT(II)	AAT(II)
31	concentrated black currant juice	yes	yes	AAT	AAT(I)	AAT(I)	AAT(I)	AAT(I)	AAT(I)	AAT(I)
32	concentrated apple juice	yes	yes	AAT	AAT(II)	AAT(II)	AAT(II)	AAT(II)	AAT(II)	AAT(II)
33	concentrated apple juice	yes	non typical	AAT	AAT(II)	AAT(II)	AAT(II)	AAT(II)	AAT(II)	AAT(II)
34	fresh apples	yes	yes	AAT	AAT(I)	AAT(I)	AAT(IA)	AAT(I)	AAT(I)	AAT(I)
35	fresh apples		yes	AAT	AAT(II)	AAT(II)	AAT(II)	AAT(II)	AAT(II)	AAT(II)
36	concentrated apple juice	yes	yes	AAT	AAT(I)	AAT(I)	AAT(I)	AAT(I)	AAT(I)	AAT(I)
37	concentrated apple juice	yes	yes	AAT	AAT(I)	AAT(I)	AAT(I)	AAT(I)	AAT(I)	AAT(I)
38	intermediate for beverage production	no	no	AAC	-	-	-	AAC(I)	AAC(I)	AAC(IA)
39	concentrated apple juice	yes	-	BG	-	-	-	BG	BG	BG
40	concentrated apple juice	yes	-	BG	-	-	-	BG	BG	BG
41	concentrated apple juice	yes	yes	AAT	AAT(II)	AAT(II)	AAT(II)	AAT(II)	AAT(II)	AAT(II)
42	concentrated apple juice	yes	yes	AAT	AAT(II)	AAT(II)	AAT(II)	AAT(II)	AAT(II)	AAT(IIA)
43	concentrated apple juice	yes	yes	AAT	AAT(I)	AAT(I)	AAT(I)	AAT(I)	AAT(I)	AAT(I)
44	spoiled apple beverage	yes	yes	AAT	AAT(II)	AAT(II)	AAT(II)	AAT(II)	AAT(II)	AAT(II)
45	concentrated apple juice	yes	yes	AAT	AAT(II)	AAT(II)	AAT(II)	AAT(II)	AAT(II)	AAT(II)
46	spoiled apple juice	yes	yes	AAT	AAT(I)	AAT(I)	AAT(I)	AAT(I)	AAT(I)	AAT(I)
47	concentrated apple juice	yes	yes	AAT	AAT(II)	AAT(II)	AAT(II)	AAT(II)	AAT(II)	AAT(II)
48	concentrated strawberry juice	no	no	AAC	-	-	-	AAC(II)	AAC(II)	AAC(II)
49	concentrated cherry juice	no	no	AAC	-	-	-	AAC(IA)	AAC(I)	AAC(IA)
50	tomato juice from fresh tomatoes	no	no	AAC	-	-	-	AAC(II)	AAC(II)	AAC(II)
51	concentrated beetroot juice	yes	yes	AAT	AAT(II)	AAT(II)	AAT(II)	AAT(II)	AAT(II)	AAT(II)
52	concentrated cherry juice	yes	yes	AAT	AAT(II)	AAT(II)	AAT(II)	AAT(II)	AAT(II)	AAT(II)
53	concentrated cherry juice	yes	yes	AAT	AAT(II)	AAT(II)	AAT(II)	AAT(II)	AAT(II)	AAT(II)
54	concentrated apple juice	yes	yes	AAT	AAT(II)	AAT(II)	AAT(II)	AAT(IIA)	AAT(II)	AAT(II)
55	concentrated cherry juice	yes	yes	AAT	AAT(II)	AAT(II)	AAT(II)	AAT(II)	AAT(II)	AAT(II)
56	concentrated raspberry juice	yes	yes	AAT	AAT(II)	AAT(II)	AAT(II)	AAT(II)	AAT(II)	AAT(II)
57	concentrated apple juice	yes	non typical	AAT	AAT(I)	AAT(I)	AAT(I)	AAT(I)	AAT(I)	AAT(I)
58	orange juice	yes	yes	AAT	AAT(II)	AAT(II)	AAT(II)	AAT(II)	AAT(II)	AAT(II)
59	concentrated apple juice	no	non typical	AAC	-	-	-	AAC(IA)	AAC(I)	AAC(I)
60	concentrated apple juice	yes	yes	AAT	AAT(I)	AAT(I)	AAT(I)	AAT(I)	AAT(I)	AAT(I)
61	concentrated apple juice	yes	yes	AAT	AAT(I)	AAT(I)	AAT(I)	AAT(I)	AAT(I)	AAT(I)
62	tomato juice	yes	yes	AAT	AAT(I)	AAT(I)	AAT(I)	AAT(I)	AAT(I)	AAT(I)
63	concentrated apple juice	yes	-	BA	-	-	-	BA	BA	BA
64	concentrated apple juice	no	no	AAC	-	-	-	AAC(II)	AAC(II)	AAC(II)
65	cherry puree	yes	yes	AAT	AAT(I)	AAT(I)	AAT(I)	AAT(I)	AAT(I)	AAT(I)
66	concentrated black currant juice	yes	yes	AAT	AAT(I)	AAT(I)	AAT(I)	AAT(I)	AAT(I)	AAT(I)
67	concentrated strawberry juice	yes	yes	AAT	AAT(II)	AAT(II)	AAT(II)	AAT(II)	AAT(II)	AAT(II)
68	cloudy apple juice	yes	yes	AAT	AAT(II)	AAT(II)	AAT(II)	AAT(II)	AAT(II)	AAT(II)
69	concentrated apple juice	yes	yes	AAT	AAT(II)	AAT(II)	AAT(II)	AAT(II)	AAT(II)	AAT(II)
70	concentrated apple juice	no	no	AAC	-	-	-	AAC(IA)	AAC(I)	AAC(I)
71	concentrated apple juice	no	no	AAC	-	-	-	AAC(IA)	AAC(I)	AAC(I)
72	concentrated apple juice	no	no	AAC	-	-	-	AAC(II)	AAC(II)	AAC(II)
73	concentrated apple juice	yes	yes	AAT	AAT(II)	AAT(II)	AAT(II)	AAT(II)	AAT(II)	AAT(II)
74	concentrated apple juice	no	no	AAC	-	-	-	AAC(II)	AAC(II)	AAC(II)
75	concentrated apple juice	no	no	AAC	-	-	-	AAC(II)	AAC(II)	AAC(II)

## Data Availability

The DNA sequences obtained during this study have been deposited in GenBank, and the accession numbers are included within the article.
